# State of the Art in the Studies on Crotamine, a Cell Penetrating Peptide from South American Rattlesnake

**DOI:** 10.1155/2014/675985

**Published:** 2014-01-15

**Authors:** Irina Kerkis, Mirian A. F. Hayashi, Alvaro R. B. Prieto da Silva, Alexandre Pereira, Paulo Luiz De Sá Júnior, Andre J. Zaharenko, Gandhi Rádis-Baptista, Alexandre Kerkis, Tetsuo Yamane

**Affiliations:** ^1^Laboratório de Genética, Instituto Butantan, Av. Vital Brasil, 1500 05503-900 São Paulo, SP, Brazil; ^2^Departamento de Farmacologia, Universidade Federal de São Paulo (UNIFESP), São Paulo, SP, Brazil; ^3^Labomar-Instituto de Ciências do Mar, Universidade Federal do Ceará, Fortaleza, CE, Brazil; ^4^Universidade Estadual da Amazônia (UEA) e Laboratório de Bioquímica e Biologia Molecular, Centro de Biotecnologia da Amazônia (CBA), Manaus, AM, Brazil

## Abstract

Animal venoms comprise a naturally selected cocktail of bioactive peptides/proteins and other molecules, each of which playing a defined role thanks to the highly specific interactions with diverse molecular targets found in the prey. Research focused on isolation, structural, and functional characterizations of novel natural biologics (bioactive peptides/proteins from natural sources) has a long way to go through from the basic science to clinical applications. Herein, we overview the structural and functional characteristics of the myoneurotoxin crotamine, firstly isolated from the South American rattlesnake venom. Crotamine is the first venom peptide classified as a natural cell penetrating and antimicrobial peptide (CPP and AMP) with a more pronounced antifungal activity. In contrast to other known natural CPPs and AMPs, crotamine demonstrates a wide spectrum of biological activities with potential biotechnological and therapeutic values. More recent studies have demonstrated the selective *in vitro* anticancer activity of crotamine. *In vivo*, using a murine melanoma model, it was shown that crotamine delays tumor implantation, inhibits tumor cells proliferation, and also increases the survival of mice engrafted with subcutaneous melanoma. The structural and functional properties and also the possible biotechnological applications of minimized molecules derived from crotamine are also discussed.

## 1. Introduction

**Figure 1:**
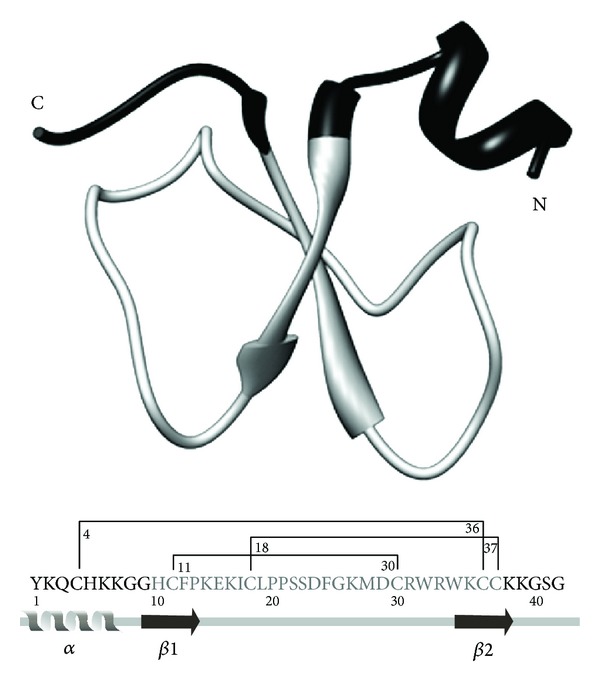
The crotamine derived peptide NrTP1 structure. NrTP1 synthetic peptide combines the N- and C-terminus of crotamine and embraces the first nine residues that are linked to the last residues 38–42, which are depicted in black in structure of the crotamine molecule above. NrTP1 and analogues are capable of membrane translocating and they localize in the nucleolus of tumor cells. Crotamine structure contains 42 residues arranged in a topology *αβ*_1_ *β*_2_: one *α*-helix with residues 1–7 and two-stranded antiparallel *β*-sheets with residues 9–13 and 34–38. The structure is stabilized by three disulfide bridges C_4_–C_36_; C_11_–C_30_; C_18_–C_37_ [1, 2]. This figure was adapted from [5].

Snake venoms contain a complex cocktail of toxins (proteins and enzymes), which are designed to assault the cardiovascular system (hemotoxic), to target specific tissues or muscle types (cytotoxic), and to go directly into the brain and nervous system (neurotoxic) of the prey. Crotamine, which is one of the major components of the venom of the South American rattlesnake *Crotalus durissus terrificus*, combines the cytotoxic and neurotoxic properties. Crotamine is a low molecular weight, nonenzymatic, and noncytolytic small protein, composed of about 42 amino acid residues. Among them, 11 are basic amino acid residues (nine lysines and two arginines), and six are cysteine residues that form three disulfide bridges [1]. These basic amino acids provide a highly positive charge for this peptide. Crotamine has two isoforms found in different subspecies, and they differ by the presence of an isoleucine residue in position 19 instead of leucine. The 3D solution structure of crotamine was determined by proton NMR spectroscopy [1], showing that crotamine structure encompasses a short N-terminal *α*-helix (involving the residues 1–7), two stranded anti-parallel *β*-sheets (residues 9–13 and 34–38), and two *β*-turns (residues 14–16 and 27–34). Consequently, it was suggested that the toxin is arranged in *β*_1_ *αβ*_2_ *β*_3_ topology, where both the first and second strands run antiparallel to the third one, while the *β*-sheet twisted in a right-handed fashion. this *β*-sheet is stabilized by four hydrogen bonds between the strands *β*_1_ and *β*_3_, (extending from the residues 10–37 and 12–35) and by other two hydrogen bonds between the strands *β*_2_ and *β*_3_ (residues 25–36). However, soon later, other authors described the presence of only two *β*-sheets [2], which is in accordance with others [3, 4]. Interchain disulfide bonds promote protein cross-linking (Cys^4^–Cys^36^, and Cys^18^–Cys^37^) between the strand *β*_3_ with the *α*-helix and the first loop (Pro^13^–Ser^23^), respectively, while the Cys^11^–Cys^30^ bond promotes the connection of *β*_1_-sheet with the second loop (Gly^26^–Trp^34^) [1, 4] (Figure 1, Table 1).

### 1.1. Genomic Structure and Chromosomal Localization of the Gene for Crotamine

In the Crotalus genus, which belongs to the Viperidae family, the karyotype is composed of 36 chromosomes: eight macrochromosome and ten microchromosome pairs. The sex determining system in this genus is heteromorphic in females (ZZ/ZW), and the fourth pair of macrochromosomes is the sex pair [7]. Fluorescent in situ hybridization (FISH) revealed that crotamine gene is localized at the end of the long arm of chromosome 2. However, the consistent difference in the intensity of the FISH signals between homologous chromosomes was evidenced, suggesting a variable number of copies of the gene on each chromosome [8]. This in turn would be a possible explanation for the variable amounts of crotamine found in the venom of crotamine-positive *C. d. terrificus* [9–11].

### 1.2. Crotamine Cytotoxicity

Toxins, by definition, are effective and specific poisonous bullets produced by the living organisms. The biological activities of crotamine were tested by intraperitoneal (IP) injection into mice in sublethal doses corresponding to 2.5 mg of toxin/kg body mass, which provokes the hind limb paralysis and the necrosis of the muscle cells of mice [9]. *In vitro,* at final concentration 10 *μ*g/mL, crotamine was demonstrated to be toxic for muscles cells, promoting muscle tissue necrosis [10, 11]. In contrast, crotamine, at final concentration ranging from 1 to 10 *μ*g/mL, was not cytotoxic to other normal cells of different types (e.g., human and mouse fibroblasts, muscle cells, human endothelial cells (HUVEC), lymphoblast (immortalized lymphocytes), mouse 3T3 cells line, mouse embryonic stem (mES) cells and others), even after 72 h of exposure [12].

### 1.3. Cell Penetrating Activity of Crotamine

Cell penetrating peptides (CPPs) are a class of bioactive molecules, also known as protein transduction domains (PTDs), membrane translocating sequences (MTSs), and Trojan peptides [13]. They are short peptides (usually ≤40 amino acid residues), with the ability to gain the access into the inner side of almost any cell [13]. Crotamine similar to other CPPs showed a rapid translocation (within 5 min) into all cell types investigated up to now [12]. Distinctly from other CPPs, crotamine demonstrates a preferential and expressive accumulation in actively proliferating cells [12]. Some CPPs show nuclear localization, as well as crotamine. Interestingly, in the nucleus, it binds to chromosomes and centrioles during the cell division. In metaphase chromosomes, fluorescently labeled crotamine binding to the chromosome produces a specific banding pattern, which is different from that presented by known DNA intercalation dyes, such as those that allow observing the G-banding or Giemsa banding and others such as the fluorescent stain 4′,6-diamidino-2-phenylindole (DAPI), which are commonly used in cytogenetic to produce a visible karyotype by staining condensed chromosomes [12]. The double helix of DNA is highly and negatively charged due to all the negatively charged phosphates in the backbone. Our recent study reported that positively charged crotamine binds noncooperatively to negatively charged DNA, covering about 5 nucleotide residues when it connects to a single or double stranded molecules [14]. Our data suggest that specific banding pattern observed on chromosomes results from electrostatic interaction between DNA phosphates and crotamine. This intrinsic biophysical property also distinguishes crotamine from other CPPs [15]. *In vivo*, crotamine penetrates into mice tissues, such as liver, skeletal muscle, bone marrow, and kidney [16]. Crotamine also crosses the blood-brain barrier (BBB), as the fluorescently labeled crotamine [16] as well as the radiolabeled form [17] was found in the brain. Three steps for the transcytosis of crotamine through the BBB can be suggested: (1) binding and internalization through endocytosis at the luminal side of endothelial cell membrane, which is negatively charged due to the presence of chondroitin and heparan sulfate molecules, (2) CPPs diffusion via the cytoplasm, and (3) externalization from endothelial cells [18, 19]. All these pathways are in accordance to the previously described mechanism of crotamine intracellular penetration [20].

### 1.4. Crotamine-Targeted Delivery of Plasmid DNA

The common feature among the CPPs is their capacity to deliver biologically active molecules into the cells both *in vitro* and *in vivo*. The majority of CPPs do not present any cell specificity for the translocation into the cells [21]. In contrast, crotamine displays specificity for actively proliferating cells, even when employed as gene delivery agent [16, 20]. This feature of crotamine could be advantageous to allow for an efficient and selective transfection of rapidly dividing cells, for example, normal stem cells, without strongly affecting the cell viability, and cancer stem cells in contrast to the currently widely used procedure, such as electroporation that causes the death of about 90% of the cells [22]. In addition, crotamine provides a successful *in vivo* transfection of bone marrow (BM) cells of mice after IP injection of crotamine-pEGFP-N1 plasmid DNA complex [16]. The proportion of BM cells displaying GFP fluorescence (about 10–20% of total) is in good agreement with the described ratio of proliferating cells present in the BM tissue. The GFP fluorescent signal was also detected in the liver and lung cells of mice [16]. In other words, the observed crotamine-mediated transfection *in vivo* [16] is similar to the previously described *in vitro* selectivity [12].

### 1.5. Mechanism of Crotamine Penetration and Cargo Delivery into Cells

Using pharmacological inhibitors and low-temperature conditions, we also demonstrated that crotamine internalization is dependent of endocytosis. Its penetration was decreased by 92.3% in the presence of chloroquine [23], which disrupts endosomal pathway by interfering with the acidification of the endosome, due to the inhibition of ion-transporting ATPase. Additionally, crotamine partially relied on the clathrin-dependent pathway for the cell uptake, since chlorpromazine (an inhibitor of clathrin mediated endocytosis) inhibited crotamine penetration by 65%. The inhibition of lipid rafts (dynamic microdomains of cholesterol, sphingolipids, and proteins clusters of the membrane) endocytosis, and also macropinocytosis did not interfere with crotamine internalization. On the other hand, low temperature (4°C) affected negatively the cell uptake of crotamine, since the efficiency of internalization could be drastically reduced showing the retention of the crotamine on the cell surface [17].

## 2. Crotamine Derived Peptides

### 2.1. CyLoP-1

The nuclear translocation process resides on lysine- and arginine-rich nuclear localization signals (NLSs). Kerkis and coworkers [12] suggested that crotamine has two putative NLS motifs, Crot_2–18_ (KQCHKKGGHCFPKEKIC) and Crot_27–39_ (KMDCRWRWKCCKK), which could be responsible for crotamine nuclear localization. The synthesis of one of such NLS motifs as thetridecapeptide Crot_27–39_ (KMDCRWRWKCCKK), which included 3 cysteine residues, and one tryptophan, aspartic acid, and methionine, along with 6 basic amino acid residues (arginine and lysine) has also been reported by Jörn Engelmann group from Germany that named this synthetic peptide as CyLoP-1 (Cytosol Localizing Peptide-1) [24]. The cell uptake of this peptide coupled to FITC presents in NIH-3T3 cell line mainly cytosol localization, instead of a nuclear distribution pattern. In order to determine the optimal length for the cell penetrating properties, single deletions of the residues from the N-terminus of CyLoP-1 have been done. Newly produced CyLoP-1 derived peptide 4 (CRWRWKCCKK) demonstrated about 20% higher cellular uptake efficiency compared to the CyLoP-1, and the best homogeneous distribution in the cell cytosol was found for this CyLoP-1 derived peptide 4. Interestingly, peptide 4 was found to be uniformly distributed in the cytoplasm along with an endosomal and vesicular fluorescence localization. The substitution of one to three cysteines by serine residue(s) led to the loss in the uptake efficiency of CyLoP-1 derived peptide 4. Based on the intracellular distribution pattern observed for this peptide, such as the perinuclear localization of the peptide, vesicles-filled fluorescent, or the cytosolic spread in different cell types, the authors concluded that, at least, CyLoP-1 derived peptide 4 uptake and its cytosolic distribution is dependent on the cell type. The carrier ability of CyLoP-1 was compared to other well-characterized CPPs, namely, Tat, penetratin, and octaarginines. D-Tat_49–57_ (RKKRRQRRR) and D-octaarginine (RRRRRRRR) and penetratin (RQIKIWFQNRRMKWKK) are also highly cationic. In addition, penetratin and CyLoP-1 derived peptide 4 (CRWRWKCCKK) are rich in hydrophobic residues. Long-term incubation of these CPPs [2.5 *μ*M] showed a lower cellular internalization in comparison to the CyLoP-1 derived peptide 4 [24]. Albeit, the same CPPs composed of D-amino acid residues showed an increased cell uptake. Indeed, the CyLoP-1 derived peptide 4, composed of L-amino acid residues was found to be the most efficient CPP for a long-term labeling [24]. Moreover, similarly to crotamine, the uptake of CyLoP-1 derived peptide 4 is strongly dependent on the cell type. Successful delivery of cargoes by CyLoP-1 also depends on their sizes. The differences in sizes and charge hydrophobicity or function of cargos in the delivery of resulting conjugates formed with CyLoP-1 derived peptide 4 were evaluated in NIH-3T3 cell line, after 18 h incubation, using the concentration of 2.5 *μ*M. As expected, the increase in molecular size resulted in an overall decrease in the intracellular uptake [24].

### 2.2. Nucleolar-Targeting Peptides (NrTPs)

It has been reported that the native crotamine also targets nucleolus, which is a nuclear structure composed by RNA, DNA, and proteins, that has different functions, such as ribosomal subunit assembly, mRNA biogenesis, and nucleolus organizer region, in immortalized and cancer cell lines, such as CHO (Chinese hamster ovary) and murine melanoma B16-F10 [16]. Rádis-Baptista and coworkers [5] designed and synthesized the nucleolar-targeting peptides (NrTPs) based on two structural simplified sequences of crotamine: Peptide 1 (YKQCHKKGGKKGSG) and Peptide 2 (YKQCHKKGGXKKGSG), both containing only one single cysteine residue (Figure 1). In HeLa cells, they were found at the nucleus and in nucleolus. Similarly to the native crotamine, these peptides bind to DNA and chromosomes at different stages of the cell cycle [15].

## 3. Crotamine Antimicrobial and Antifungal Activity

The antimicrobial peptides (AMPs) are small cationic peptides, responsible for the adaptive immunity in the external surface of skin and mucus of several organisms across the evolutionary spectrum [25]. The AMPs are a unique class composed of varying molecules grouped into subgroups based on their amino acid composition and structure. AMPs are classified into three major groups: (i) peptides with an *α*-helical conformation (insect cecropins, magainins, etc.), (ii) cyclic and open-ended cyclic peptides with pairs of cysteine residues (defensins, protegrin, etc.), and (iii) peptides with an overrepresentation of some amino acids (proline-rich, histidine-rich, etc.) [26]. AMPs present a rapid killing and broad-spectrum antimicrobial activities [27]. AMPs are usually composed of 12 to 50 amino acid residues, and they show at least two or more positively charged residues, generally represented by arginine, lysine, or histidine, in acid environments, and with a high content of hydrophobic residues (usually >50%) [28]. Moreover, most AMPs display hydrophobic and cationic properties, have a molecular mass below 25–30 kDa, and adopt an amphipathic structure (alpha-helix, beta-hairpin-like beta-sheet, beta-sheet, or alpha-helix/beta-sheet mixed structures) [29]. The ability to associate with the cell membrane is a key feature of the AMPs, although the membrane permeabilization is not an essential requirement for their activity [30]. Different peptides act in different ways and the exact mechanisms are only beginning to be elucidated. Some intracellular targets have also been described [31]. In fact, speculations that transmembrane pore formation is not the only mechanism of microbial killing suggest that translocated peptides can alter cytoplasmic membrane septum formation, inhibit cell-wall synthesis, inhibit nucleic acid synthesis, inhibit protein synthesis, or inhibit enzymatic activity [30]. It seems that crotamine is also a potential candidate to be included in this class of compounds with antimicrobial activities [32]. More interestingly, it is likely that crotamine and the well-known AMP defensin could have been derived from a common ancestor gene [33]. This was suggested by Nicastro and coworkers [1], who first described the similarities of the disulfide bonds pattern of these molecules. However, significant differences in the primary structure/amino acid composition are observed for these molecules [34]. Despite the well-known broad antimicrobial spectrum of defensins [35], crotamine shows a modest activity towards both Gram-negative and Gram-positive bacteria [36].

Yount and collaborators [33] also suggested the potential antibacterial and antifungal activity of crotamine. A more careful determination of minimal inhibitory concentration (MIC) of crotamine towards a broad range of microorganisms was only recently determined by Mirian Hayashi's group [36]. Crotamine's important activity against several fungi of *Candida* spp., in particular *C. albicans*, including against clinical resistant strains, was observed [36]. It is of note that this Candida species are among the most common bloodstream pathogens in the United States and rank seventh among etiologic agents in Europe [37], and the pronounced effective antifungal activity observed by our group for crotamine opens new perspectives for the use of this venom component in biomedicine, more specifically for infectious disease treatments [36]. Moreover, some AMPs are also found to possess translocating activity across the cell membrane, which can interfere with critical cellular functions leading to cell death [38]. Similar apoptotic activity in combination with cell penetration was described for crotamine by our group for several cell types [39]. Moreover, the AMPs are thought to have other polyanions, such as DNA or RNA, as their ultimate target [40].

## 4. Crotamine Anticancer Cells Activity

Actually, it is well accepted that the majority of antimicrobial compounds might also have antitumor activity [41]. It could be simply due to the 3D structural similarities or even due to an evolutionary relationship, which still remains to be determined [42]. The cationic feature might also be determinant of both activities [43]. The electrostatic interactions between the negatively charged components of the membrane of cancer cells and the positively charged peptide are believed to play a major role in the strong binding of the peptide and its ability to selectively disrupt the membrane of cancer cells [44].

Investigation of crotamine anticancer cells toxicity and their *in vitro* and *in vivo* efficacy in mouse model of melanoma was examined by Kerkis's group [45, 46]. This toxin at concentrations of 1–5 *μ*g/mL was used to test the viability of B16-F10 (murine melanoma), SK-Mel-28 (human melanoma cells), and Mia PaCa-2 (human pancreatic carcinoma cell line). The nonmalignant neoplastic murine fibroblasts 3T3 cell line was used as control. Interestingly, that crotamine at final concentration of 5 *μ*g/mL was lethal to B16-F10, Mia PaCa-2, and SK-Mel-28 cells, while it was inoffensive to normal cells [45]. Additionally, we showed that differently from several anticancer drugs, crotamine targets primary lysosomes and mitochondria, leading to increases of intracellular free calcium concentrations in cancer cells [16]. Once the balance of cellular uptake and efflux determines drug accumulation, we also measured the crotamine permanence in cancer cells. Using Cy3-crotamine, which was added to B16-F10 cells, it was possible to observe that at least 70% of the cells hold the fluorescence signal during approximately 20 h, suggesting a long-term retention of crotamine in these cells [45]. *In vivo*, in mouse model for melanoma, crotamine demonstrates selective penetration into tumor melanoma cells, predominantly observed within the cells in tumor masses, in the cells around the tumor necrotic areas, and in rapidly dividing metastatic cells, but not in normal cells surrounding the tumors [39, 45].

Fluorescent crotamine traces metastatic invasion of B16-F10 cells, suggesting its possible applications as an imaging agent and metastasis marker in living organisms [39].

Delay of melanoma tumors implantation was also observed after 21 days of chronic treatment with crotamine (1 *μ*g per animal, per day), in a mice model that received B16-F10 cells (10^5^ cells) by subcutaneous injection. This treatment, started at the first day after melanoma cells injection, allowed to observe that crotamine significantly inhibits the tumor growth, as evaluated by measuring the tumor mass weight, and also prolongs the lifespan of these mice bearing B16-F10 tumor [45]. Histological examination also confirmed that crotamine is nontoxic to normal cells and normal tissues, such as kidney and liver, at the used concentrations, as well as that crotamine does not demonstrate any immunotoxic effect after *in vivo* long-term treatment of mice bearing melanoma tumors [39, 45]. The low immunogenicity of some snake venom toxins is a well-known feature, which in the case of crotamine could be attributed to its small size [46].

More recently, using noninvasive optical imaging procedure that permits *in vivo* real-time monitoring of fluorescent molecules uptake, crotamine localization into remote subcutaneous tumors engrafted in nude mice was confirmed [39]. Additionally, this study also demonstrated that the inhibition of tumor growth involves mitochondrial depolarization and intracellular calcium release. These data indicated that the cytotoxic peptide crotamine could potentially be used for a dual purpose: to target and detect growing tumor tissues and also to selectively trigger tumor cell death [39].

Interestingly, infections caused by *Candida* spp. are frequent and serious in oncology patients [47]. Definitely, a more precise evaluation of the structure-function relationship for both antimicrobial and antitumor activity of crotamine might allow for the discovery and proposal of potential novel mechanisms or of new structural models for the treatment of cancer and/or for the development of a more selective antimicrobial compounds that might not act on mammals host cells. Comparative studies to further understand the determinants of antimicrobial and antitumor activities of crotamine are considered by our group to be highly significant.

### 4.1. Crotamine-Like Peptides

Crotamine shares similar structure features with the defensins, which are a family of small cysteine-rich cationic proteins found in both vertebrates and invertebrates. Similarly to crotamine, defensins (from vertebrates) present a compact structure and consist of 18–45 amino acid residues, including the presence of six to eight conserved cysteine residues [48]. Biologically active crotamine-like peptides (CLPs) were also described in the venom of some sauropsid reptiles (lizards and snakes), scorpions (*Scorpio maurus palmatus*), and in small mammals such as Platypus (*Ornithorhynchus anatinus*), a semiaquatic mammal from Eastern Australia [49]. The mechanism of action of CLPs is very wide, including targets as the voltage-gated Na^+^ (Na_v_s) [50], K^+^ (K_v_s) [51], and acid-sensing ion channels (ASICs) [52]. Crotamine was also supposed to present similar effect. In fact, Peigneur et al. [53] reported that crotamine potently and selectively blocks mammalian voltage-gated K_v_1.3, although it was also demonstrated that crotamine does not act on Na_(v)_ channels [54]. This was the first study, which shows a neurotoxin from the snakes of Crotalus genus targeting an ion channel.

## 5. Conclusions

Indeed, learning more about the way cancer cells is different from normal cells will allow us to identify and target specific proteins in cancer cells. Any new treatment proposal aims to be more efficient and with fewer side effects. Since cancer cells hardly differ physiologically from healthy cells, anticancer drugs do not specifically affect only cancer cells, but mistakenly also affect all other normal dividing cells. Compared to other known anticancer targeted drugs, only crotamine was shown to have the ability to selectively target actively proliferating living cells, both *in vitro* and *in vivo*, without visible effect on normal dividing cells.

Similarly to AMPs, crotamine is highly soluble in water and is capable of interacting strongly with biological membranes. This might be due to its N-terminal polyarginine domain, which may allow its interaction with negatively charged phosphate moieties in the cell membranes, which is suggestive of a cell-penetrating domain [55]. Crotamine is also selectively attracted to negatively charged microbial membranes [36]. Additionally, it presents significant antifungal and antitumor activity [36]. Inside the several cell types, crotamine interacts with secondary targets, thus being able to interrupt vital metabolic processes [16]. Moreover, the recent findings by Peigneur et al. [53] demonstrated that crotamine selectively blocks mammalian K_v_1.3, heterologously expressed in *Xenopus laevis* oocytes. It also sheds more light on the possible role of voltage-gated potassium channels blockage in its anticancer effect, but more studies are still required to clarify it.

**Figure 2:**
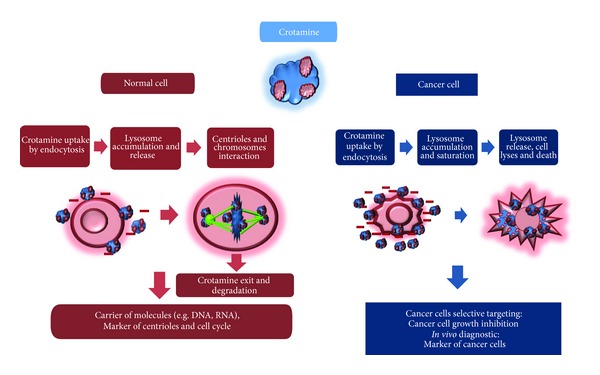
Crotamine action in normal (red) and cancer (blue) cells. Crotamine is a positively charged (blue) protein. In normal cells, crotamine uptake occurs through clatrin-dependent endocytosis followed by lysosome accumulation, followed by its release in the cytosol due to the disruption of the vesicles containing crotamine. In normal cells, crotamine interacts electrostatically with centrioles and chromosomes and can be used as a biotechnological tool, as carrier of bioactive molecules, and as a marker of cell cycle. Cancer cells, which have more negatively charged molecules on their surface compared to normal cells, potentially attract crotamine strongly. In cancer cells, crotamine intracellular concentration appears to be higher than that observed in normal cells, thus probably leading to cell lyses and cell death. In cancer cells, crotamine inhibits tumor growth and kills tumor cells, besides representing a potential tool for *in vivo* cancer cells identification.

In Table 1, we summarized the possible biotechnological applications of crotamine and their derivatives. We demonstrate that crotamine acts differently in normal actively proliferating (dividing) and cancer cells (Figure 2). This study provides new insights, which may contribute to unveil the differences among the normal and cancerous cells using as a tool the crotamine, which is a versatile and multifunctional peptide, as described here. On the other hand, crotamine and their derivates represent already a potential biotechnological tool. Further studies need to become crotamine derivates marketable. Although, they are potentially ready to be used both *in vitro* and *in vivo* as molecular carrier, marker of cell cycle and of centrioles, and as biomarker of cancer cells.

## Figures and Tables

**Table 1:** Crotamine main characteristics, properties, and potential biotechnological applications*.

Name	Crotamine
Organism	*Crotalus durissus terrificus* South American rattlesnake Common name: Cascavel
Taxon authority	[6]
Geographic range	Brazil, Peru, Bolivia, Paraguay, Uruguay, Argentina*
Classification	Myoneurotoxin, cell-penetrating peptide, antimicrobial peptide, and defensin-like peptide
Molecular weight	4726,63 daltons
Isoelectric point	pI 9.54, highly positive
Length	Polypeptide of 42 amino acids
Primary sequence	KQCHKKGGHCFPKEKICLPPSSDFGKMDCRWRWKCCKKGSG
Isoform	Crotamine-Ile 19 (isoleucine substitution at position 19)*
Chemical formula	C_346_H_530_N_90_O_82_S_8_ (isoform 1)*
Solubility	Highly soluble in water and physiological solutions
Stability	Highly stable in solution, relative large pH range, and temperature
Folding	Crotamine is arranged in a *αβ*_1_ *β*_2_ topology stabilized by 3 disulfide bridges: an *α*-helix with residues 1–7 and a two-stranded antiparallel *β*-sheets with residues 9–13 and 34–38.
Disulfide bonds	C_4_–C_36_; C_11_–C_30_; C_18_–C_37_
Physiological and neurological activity	Hind limb paralysis in mice in final concentration 2.5 mg of toxin/kg body mass Necrosis of muscle cells
Electrophysiology activity	Mammalian K_v_1.1, K_v_1.2, and K_v_1.3 blocker with IC_(50) _of 286.53 ± 91.72 nM
*In vitro* toxicity (normal cells)	Nontoxic (concentration ranged from 0.1 to 10 *μ*M)
Embryotoxicity	Nontoxic (concentration ranged from 0.1 to 10 *μ*M)
Cell penetrating activity (*in vitro*)	Selective: dividing (actively proliferating cells) Concentration, cell type, and cell-cycle dependent
Intracellular localization	Cytosol, vesicles, nucleus, centrioles, and chromosomes
Mechanism of DNA-crotamine interaction	Only electrostatic: crotamine (+charged)—DNA (−charged) aggregate
Uptake	Within 5 minutes, and permanence in the cells for approximately 24 hours
Mechanism of penetration	Membrane heparan sulfate proteoglycans binding and clathrin-dependent endocytosis
Cell penetrating activity (*in vivo*)	Selective: dividing (actively proliferating cells), for example stem cells in bone marrow, spleen, liver, lung, and so force.
Localization in brain	Able to cross blood-brain barrier, and localization in brain cells
Molecular carrier	Intracellular delivery of DNA (both circular and linear molecules) *in vivo* and *in vitro. *Final complex size dependent delivery.
Antimicrobial activity	Modest against Gram-positive and Gram-negative bacteria, with some exceptions, for example *Micrococcus luteus*, and with no detectable activity against the filamentous fungus *Aspergillus fumigatus* and *Trichophyton rubrum* at concentrations up to 125 *μ*g/mL.
Antimycotic (-fungal) activity	Significant activity against yeast *Candida* spp.
Cancer cells toxicity	Toxic (concentration ranged from 0.1 to 10 *μ*M) Inoffensive for normal cells
Anticancer activity (*in vitro) *	Strong against melanoma cells *in vitro *
Anticancer activity (*in vivo*)	Inhibition and delay of melanoma growth *in vivo* in mouse model
Mechanism of tumor inhibition	Mitochondrial depolarization Intracellular calcium release
Immunogenicity	Low
Biotechnological and biomedical applications	Marker of centrioles and cell cycle; marker of actively proliferating normal cells; biomolecules carrier; tool for cancer cells investigation; marker of cancer cells *in vitro* and *in vivo*, and as antifungal and anticancer agent. Prototype for new drug design.

*Several old data about crotamine need to be revised using modern approaches.
